# Sex‐Specific Phenotype‐Performance Links: Divergent Correlations Between Morphology, Coloration, and Bite Force in the Mountain Dragon (*Diploderma vela*)

**DOI:** 10.1002/ece3.73071

**Published:** 2026-02-11

**Authors:** Songwen Tan, Ling Li, Wei Gao, Guocheng Shu, Peng Guo, Yayong Wu

**Affiliations:** ^1^ Faculty of Agriculture, Forestry and Food Engineering Yibin University Yibin China; ^2^ State Key Laboratory of Genetic Evolution and Animal Models, Yunnan Key Laboratory of Biodiversity and Ecological Conservation of Gaoligong Mountain, Kunming Institute of Zoology Chinese Academy of Sciences Kunming China

**Keywords:** bite force performance, body coloration, mountain dragon, phenotypic traits, sexual dimorphism

## Abstract

Sexual dimorphism in lizards arises from the dynamic interplay between natural and sexual selection, manifesting in divergent phenotypic traits across taxa. A key unresolved question is whether the relationship between such sexually dimorphic traits and functional performance also differs between the sexes. This study investigated this question in the mountain dragon (*Diploderma vela*), a protected species endemic to the upper Lancang River basin in southwestern China, by quantifying its sexual dimorphism in morphology and coloration and assessing their sex‐specific correlations with bite force. A total of 94 individuals were assessed for nine morphological traits, maximum bite force capacity, and body coloration across 15 anatomical regions. After controlling for body size, significant male‐biased dimorphism was detected in most morphological traits, whereas abdomen length was female‐biased. Coloration also differed between sexes across all measured regions except the abdomen. Crucially, the relationship between morphology and bite force was sex‐specific; different suites of traits predicted bite force in males versus females. In contrast, no correlation was found between coloration and bite force in either sex. These divergences reflect the species' flexible phenotypic responses to varying reproductive and ecological pressures. These findings demonstrate that sexual dimorphism extends beyond trait means to encompass sex‐specific phenotype‐performance relationships, highlighting differential adaptive responses. This work provides a functional framework for understanding trait evolution in *D. vela* and underscores the need for sex‐specific considerations in its conservation.

## Graphical abstract

*D. vela* exhibited pronounced sexual divergence in morphological traits, bite force, and body coloration, shaped by the interplay of three evolutionary mechanisms: sexual selection, fecundity advantage, and intraspecific niche divergence. These complementary, non‐exclusive processes collectively enhance adaptive fitness in the hot, dry river valley ecosystems. By enabling flexible phenotypic responses to both reproductive and ecological pressures, this trait modularity may bolster population persistence under intensifying climate‐driven aridification and environmental stochasticity.

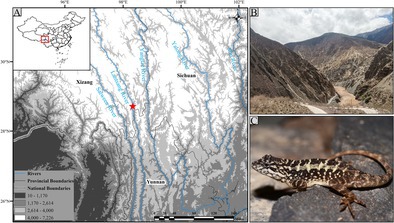

## 1 Introduction

Sexual dimorphism, defined by divergent phenotypic traits between males and females of the same species, is a widespread evolutionary outcome observed across diverse animal taxa, including invertebrates, amphibians, reptiles, birds, and mammals (Pinto et al. 2005; Jozet‐Alves et al. 2008; Zhong et al. 2017; Andre et al. 2018; Pincheira‐Donoso et al. 2020). Despite its ubiquity, the expression of dimorphic traits is remarkably heterogeneous, with patterns varying not only across higher taxonomic levels but also within families and genera, particularly in reptilian clades (Cox et al. 2009; Cruz‐Elizalde et al. 2021). These dimorphic patterns are manifested through multiple phenotypic dimensions, including body size and coloration in reptiles (Herrel et al. 2018; Pincheira‐Donoso et al. 2020; Stuart‐Fox et al. 2020).

Body size is commonly employed as a principal indicator of sexual dimorphism, with sexual size dimorphism (SSD) representing one of the most pervasive signatures of sex‐specific adaptive divergence across reptiles (Fairbairn et al. 2007; Pincheira‐Donoso et al. 2020). Three primary SSD patterns have been identified within reptiles (Cruz‐Elizalde et al. 2021), including male‐biased (Herrel et al. 2010), female‐biased (Cox et al. 2009), and unbiased (Schwarzkopf 2005). However, reliance on size metrics alone may obscure finer‐scale phenotypic differentiation. Morphological traits such as cephalic proportions and appendicular dimensions often yield finer resolution in characterizing sex‐specific divergence (Cruz‐Elizalde et al. 2020). Notably, these specific morphological traits are often directly linked to functional performance, thereby exerting a direct influence on an individual's survival and adaptation (Li et al. 2025).

Body coloration serves as another primary axis of sexual dimorphism in reptiles. The recurrent and phylogenetically independent evolution of conspicuous sexual coloration suggests that coloration plays important adaptive roles (Stuart‐Fox et al. 2020). However, most investigations into sexual dimorphism in reptile coloration remain restricted to descriptive comparisons of conspicuous integumentary patterns, with few studies quantifying specific color traits such as brightness and chroma (Qiu et al. 2022). Previous research has demonstrated that brightness in lizard coloration is strongly correlated with ecological processes such as thermoregulation and camouflage (Smith et al. 2016), whereas chroma is potentially associated with male–male competition (Bajer et al. 2011). Moreover, the lack of comprehensive quantitative measurements across all body regions precludes a precise assessment of whether significant sexual differences exist in areas lacking prominent markings, particularly those critical for signal communication and social displays (de Pérez I Lanuza et al. 2013). Therefore, an integrated analysis incorporating specific morphological traits and quantitative color metrics across multiple anatomical regions is imperative to fully elucidate the multifaceted nature of sexual dimorphism in reptiles (Zhang et al. 2018).

A key unresolved question concerns the adaptive significance of these dimorphic traits. While numerous studies have documented patterns of morphological and chromatic dimorphism in reptiles (Xiong et al. 2022; Li et al. 2025), fewer have explored their adaptive functional implications (Pincheira‐Donoso and Hunt 2017). Phenotypic traits often serve as reliable indicators of functional performance in reptiles (Xiao et al. 2024), for instance, external morphological features can effectively reflect bite force capacity in lizards (Isip et al. 2022). However, it remains poorly understood whether, in sexually dimorphic species, males and females utilize the same phenotypic traits to signal their functional performance (e.g., bite force), which is crucial for survival and reproduction in similar behavioral contexts (Isip et al. 2022). Namely, whether the correlation between phenotype and performance is itself sexually dimorphic requires further investigation.

*Diploderma vela*, an agamid lizard described in 2015, is endemic to the arid upper Lancang River valley of southwestern China, where it occupies rocky outcrops and xeric shrublands, and feeds primarily on insects and spiders (Wang et al. 2015). Due to its restricted distribution and strong habitat specificity, the species was designated a National Second‐level Protected Wild Animal in China in 2021. However, ongoing hydropower development has resulted in extensive habitat degradation, driving severe population declines. Despite its conservation priority, core biological and ecological data for *D. vela* remain limited.

Preliminary field observations suggested conspicuous sexual dimorphism in body size and coloration in *D. vela*. To move beyond qualitative description, this study systematically tested two main hypotheses: (1) that *D. vela* exhibits pronounced sexual dimorphism in both morphological traits and body coloration across various anatomical regions, and (2) that the relationships between these phenotypic traits and a key performance measure (maximum bite force) are sex‐specific. By establishing foundational data on functional morphology, this study enhances the biological profile of the species and provides valuable insights for conservation strategies. Furthermore, the analytical framework developed here may inform studies of sexual dimorphism in other habitat‐specialized reptiles.

## 2 Materials and Methods

### 2.1 Study Site

**FIGURE 1:**
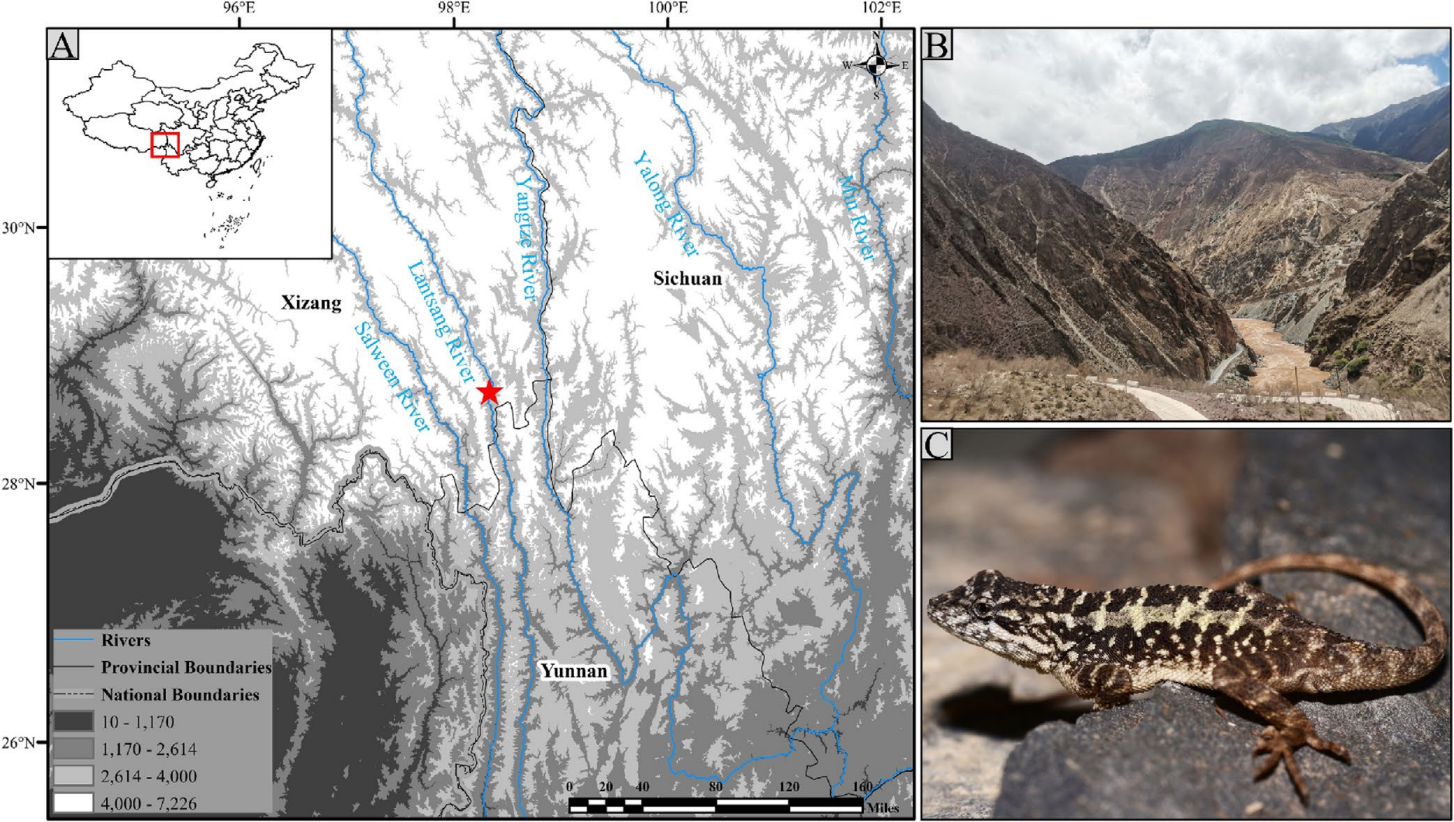
Study site (A), habitat (B), and male individual (C) of *D. vela*. Red star represents the research site.

Fieldwork was conducted in Quzika Township (29°05′ N, 98°36′ E; 2350 m a.s.l.), located in the upper Lancang River valley of Mekong County on the eastern Tibetan Plateau, China (Figure 1). This site represents the type locality of *D. vela* and harbors the highest known population density. Individuals were primarily observed inhabiting rock crevices and shrub‐steppe vegetation along valley slopes adjacent to the Lancang River. The region has a continental monsoon climate, typified by hot, humid summers and cold, dry winters. Annual precipitation ranges from 350 to 450 mm, with most rainfall occurring between June and September. A brief frost‐free period of approximately 95 days, coupled with pronounced seasonality, defines a habitat that also sustains potential predators such as the copperhead racer (*Elaphe taeniura*) and common kestrel ( *Falco tinnunculus* ) (Wang et al. 2015).

### 2.2 Animal Sampling

A total of 94 *D. vela* individuals (57 males and 37 females) were captured in June 2020 using manual and noose techniques during peak diurnal periods (10:00–17:00). Each specimen was assigned a unique identification code (marked on the exterior of the container), with sex and precise geographical coordinates recorded at the time of capture. Sex determination was based on dorsal coloration pattern and the presence or absence of hemipenal bulges. Morphological traits were recorded on‐site. Subsequently, coloration and bite force were measured under standardized indoor conditions, and all individuals were released unharmed at their original capture locations within 2 days. All procedures were conducted in accordance with ethical guidelines approved by the Animal Care Committee of Yibin University (License No. YBU2020008) and with its permission to ensure animal welfare and ecological integrity.

### 2.3 Measurement of Morphology Traits, Bite Force, and Body Coloration

Morphological traits were quantified using a precision digital caliper (Deli DL91200, Ningbo, China; accuracy ±0.01 mm) and an electronic balance (Mengfu I‐2000, Dongguan, China; accuracy ±0.01 g). Nine linear variables were measured following established protocols (Cruz‐Elizalde et al. 2020), including snout‐vent length (SVL), tail length (TL), abdomen length (AL), head length (HL), head width (HW), head height (HH), mouth length (ML), forelimb length (FLL), and hindlimb length (HLL). All measurements were performed by a single investigator to avoid errors, with three replicates per trait used to calculate means.

Cloacal body temperature was recorded using an ultra‐thin catheter electronic thermocouple (Victor UT323, Shenzhen, China; resolution +0.01°C), inserted 5 mm into the cloaca. Bite force was assessed using a piezoelectric force transducer (Viste VXT500, Shenzhen, China; 0–50 N), composed of a pair of 2‐mm‐thick steel bite plates (resolution 0.1 N), a hand‐operated amplifier, and a digital output display. To prevent dental injury, each bite plate was fitted with a layer of non‐toxic rubber. Prior to each session, the system was calibrated using certified weights. A 30‐min acclimatization period under standardized thermal and lighting conditions preceded measurements to ensure lizards were metabolically active and their color expression was consistent. During trials, lizards were gently restrained and positioned with the rostrum aligned perpendicularly to the sensor surface to ensure full jaw engagement. Three bite trials were performed under controlled thermal conditions. Following standard protocols (McBrayer and Anderson 2007), the maximum bite force across all trials, along with its corresponding body temperature, was used for analysis.

**FIGURE 2:**
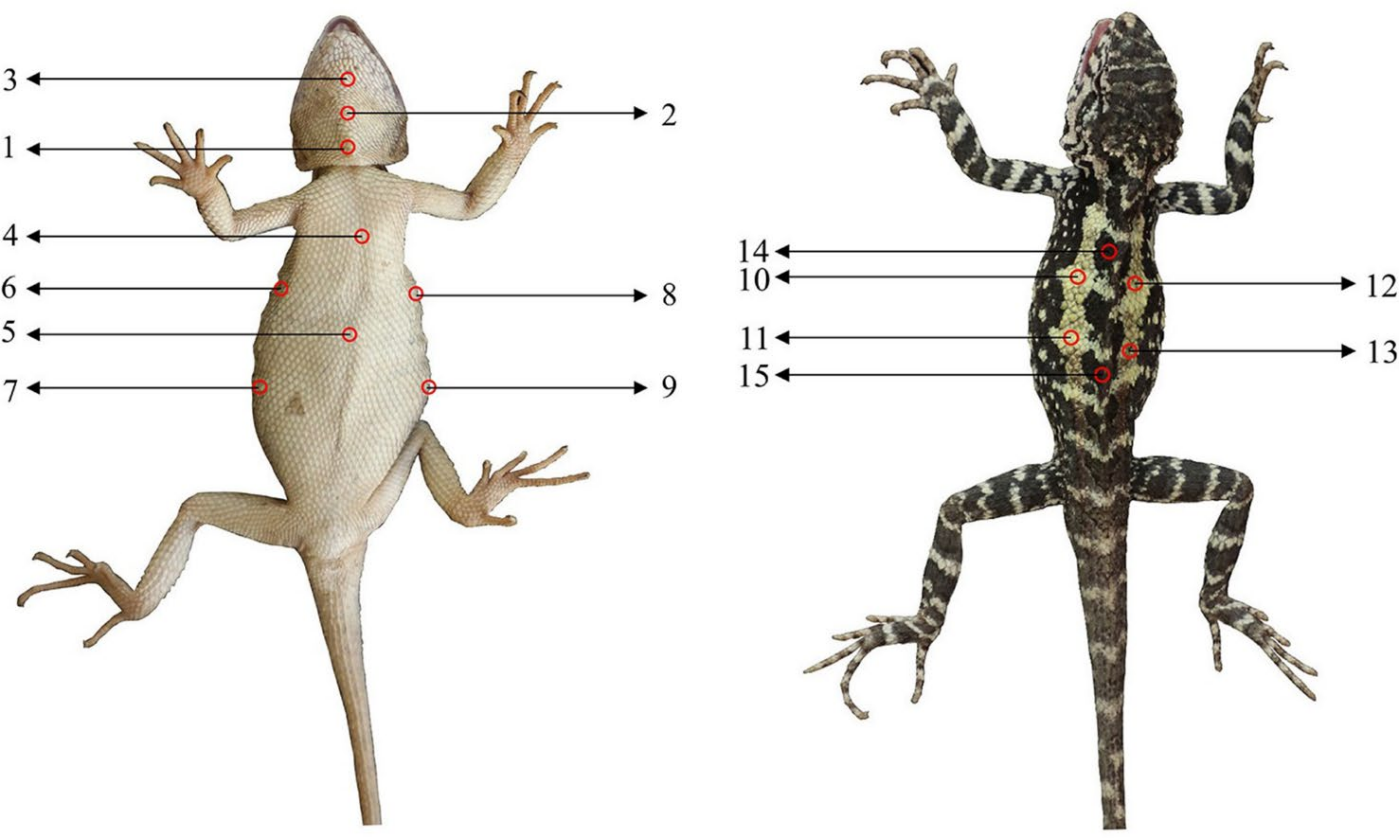
Schematic of body coloration measurement sites in *D. vela*. Spectral data were collected from identical anatomical locations in both male and female individuals. 1–3: Throat; 4–5: Abdomen; 6–9: Lateral torso; 10–13: Dorsal light markings; 14–15: Dorsal dark markings.

Body coloration was measured using an AVASPEC‐2048 fiber‐optic spectrometer (Ocean Insight, USA) equipped with a halogen light source and a reflection probe. Spectral measurements were taken from 15 points distributed across five anatomical regions: throat (3 points), abdomen (2 points), lateral torso (4 points), dorsal light markings (4 points), and dorsal dark markings (3 points) (Figure 2). The probe was held perpendicular to the skin surface at a fixed distance of 2 mm, with reflectance spectra (300–700 nm) acquired at a 100 ms integration time using SpectraSuite software. Spectral data were categorized into biologically relevant wavelength bands: ultraviolet (UV, 300–400 nm), blue (400–475 nm), green (475–550 nm), yellow (550–625 nm), and red (625–700 nm). Triplicate measurements were taken at each point to ensure reproducibility, with irradiance calibration performed to reduce environmental variability.

### 2.4 Coloration Parameter Calculation

To investigate whether detailed body color traits exhibit sexual dimorphism, reflectance spectra, including luminance and chroma components, were collected. The formula for luminance is as follows:

$$\mathrm{Qt}=\sum R\left(\lambda \right)$$

where *R* is reflectance at specific wavelength λ and *Qt* represents total reflectance from 300 to 700 nm.

Relative luminance for each color band was subsequently calculated using the following formulae:

U=Quv/Qt

B=Qb/Qt

G=Qg/Qt

Y=Qy/Qt

R=Qr/Qt

where *U*, *B*, *G*, *Y*, and *R* represent the relative luminance of UV, blue, green, yellow, and red light, respectively, and *Quv*, *Qb*, *Qg*, *Qy*, and *Qr* represent the reflectance ratio of UV, blue, green, yellow, and red light, respectively.

Chroma (*C*) was then calculated as:

$$C=\sqrt{{\left(R-G\right)}^{2}+{\left(Y-B\right)}^{2}}=\sqrt{{\mathrm{LM}}^{2}+{\mathrm{MS}}^{2}}$$

where LM represents the relative difference in reflectance between red and green light, and MS represents the relative difference in reflectance between yellow and green light.

### 2.5 Statistical Analysis

All data were logarithmically transformed to approximate normality, with missing values imputed using multiple imputation by chained equations (MICE). Assumptions of normality (Shapiro–Wilk test) and homoscedasticity (Levene's test) were verified, applying Box‐Cox transformation when violations occurred.

Sex‐based differences in morphological traits and bite force were initially evaluated using one‐way analysis of variance (ANOVA). Pearson correlation analyses were then conducted between SVL and other morphological traits for males and females. If significant positive correlations with SVL were observed across traits, multivariate ANOVA (MANCOVA) was employed to assess sex differences in morphology and bite force, using SVL as a covariate. To test whether morphological traits exhibit sexual dimorphism in allometry, Analysis of Covariance (ANCOVA) was used to examine the significance of the interaction term (log_10_SVL × Sex), thereby determining whether the allometric slopes differ between the sexes.

For body coloration, mean spectral reflectance was calculated for each anatomical region, and reflectance curves were generated accordingly. Sex‐specific differences in luminance and chroma across anatomical regions were tested using independent samples *t*‐tests.

To investigate potential sex‐specific differences in the correlations between phenotypic traits (morphology and coloration) and functional performance (maximum bite force) in *D. vela*, Spearman correlation analyses between all phenotypic traits and maximum bite force were first conducted. Subsequently, traits exhibiting significant correlations were selected as predictor variables to construct random forest (RF) models, with maximum bite force defined as the response variable. Compared to conventional linear regression, RF models more effectively mitigate the influence of correlations among predictor variables (multicollinearity). Based on the Mean Decrease Gini index from the RF models, the four most important predictors for each sex were identified. Finally, the relationships between these primary predictors and bite force were graphically illustrated using least squares polynomial fitting.

All statistical analyses were conducted using R v4.2.2 and SPSS v27.0. Visualizations were generated using the R package “ggplot2” and Origin 2024. Statistical significance was set at *p* < 0.05.

## 3 Results

### 3.1 Sexual Dimorphism in Morphology

**FIGURE 3:**
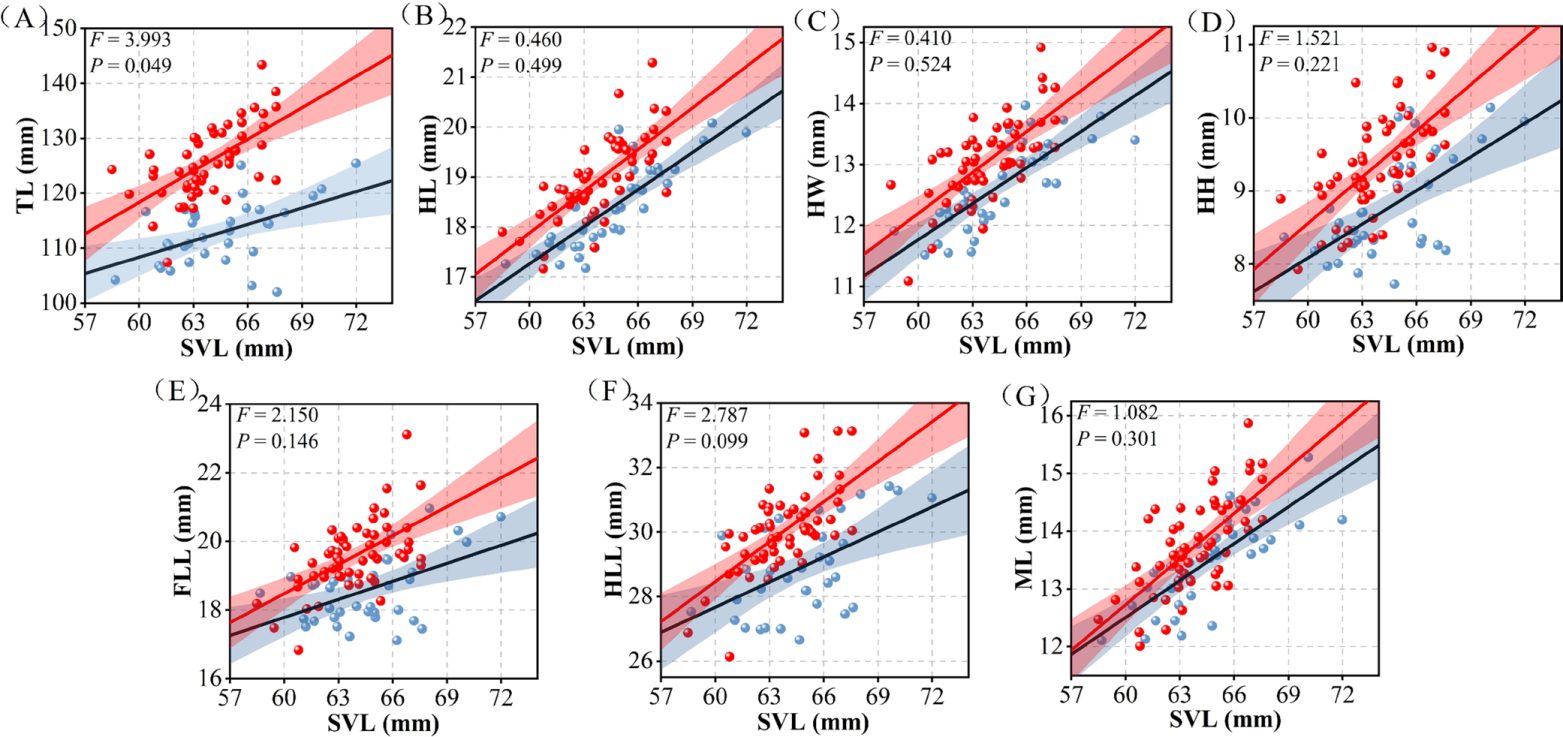
Comparison of growth rates in morphological traits between males and females. Red represents males, black represents females. Abbreviations can be found in the 2. Materials and Methods section.

Significant sexual dimorphism was observed between males and females across multiple morphological traits (Table 1). Although one‐way ANOVA revealed no significant differences in SVL (*F* = 2.502, *p* = 0.117) and ML (*F* = 2.004, *p* = 0.160), results of the MANCOVA, with SVL as a covariate, showed that males possessed significantly larger trait values than females for TL (*F* = 155.361, *p* < 0.001), HL (*F* = 46.235, *p* < 0.001), HW (*F* = 28.813, *p* < 0.001), HH (*F* = 39.047, *p* < 0.001), ML (*F* = 12.892, *p* < 0.001), FLL (*F* = 40.312, *p* < 0.001), and HLL (*F* = 39.052, *p* < 0.001). In contrast, abdomen length was significantly greater in females (*F* = 52.410, *p* < 0.001). Regarding growth rates, TL exhibited allometric growth between sexes (*F* = 3.993, *p* = 0.049), whereas other traits conformed to isometric growth patterns (Figure 3).

**TABLE 1:** Mean values of morphological traits between males and females.

Morphological traits	Male (*n* = 57)		Female (*n* = 37)		ANOVA		MANCOVA	
	Mean ± SD	*R*	Mean ± SD	*R*	*F*	*p*	*F*	*p*
SVL (mm)	63.83 ± 2.07	1.000*	64.66 ± 2.82	1.000*	2.502	0.117	/	/
TL (mm)	125.69 ± 6.49	0.507*	112.92 ± 5.78	0.619*	94.96	< 0.001	155.361	< 0.001
AL (mm)	29.99 ± 1.30	0.856*	31.80 ± 1.88	0.819*	28.75	< 0.001	52.410	< 0.001
HL (mm)	18.95 ± 0.79	0.840*	18.42 ± 0.83	0.727*	9.757	0.002	46.235	< 0.001
HW (mm)	13.06 ± 0.67	0.793*	12.69 ± 0.70	0.687*	6.496	0.013	28.813	< 0.001
HH (mm)	9.36 ± 0.67	0.624*	8.80 ± 0.69	0.647*	15.69	< 0.001	39.052	< 0.001
ML (mm)	13.74 ± 0.80	0.777*	13.50 ± 0.77	0.680*	2.004	0.160	39.052	< 0.001
FLL (mm)	19.56 ± 1.00	0.508*	18.60 ± 0.97	0.583*	21.26	< 0.001	39.052	< 0.001
HLL (mm)	30.04 ± 1.28	0.532*	28.88 ± 1.37	0.666*	17.42	< 0.001	39.052	< 0.001

*Note:* Abbreviations can be found in the 2. Materials and Methods section.

* Significant correlation (*p* < 0.05).

### 3.2 Sexual Dimorphism in Body Coloration

**FIGURE 4:**
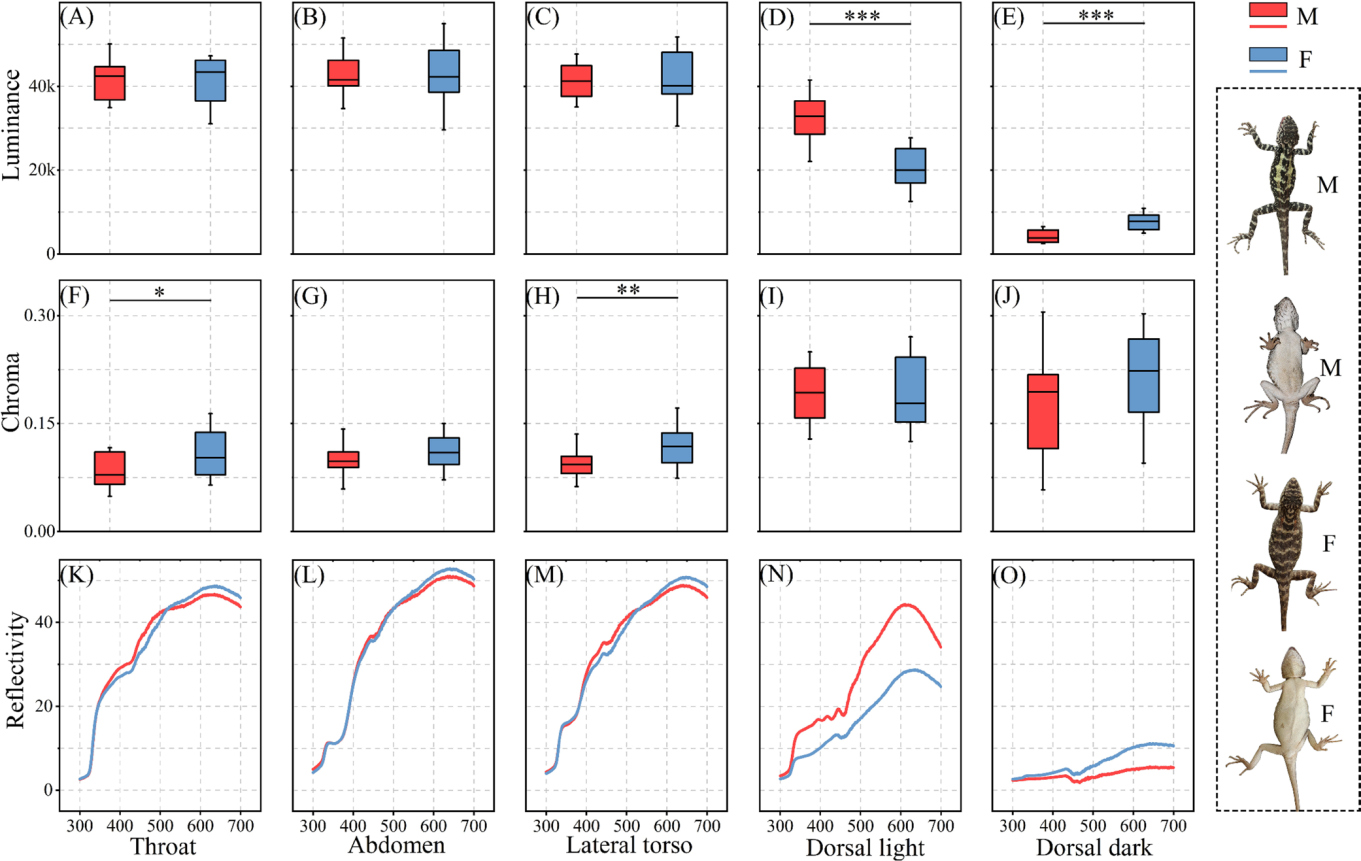
Sexual body coloration dimorphism in *D. vela*. *Significant difference (*p* < 0.05), **Very significant difference (*p* < 0.01), ***Extremely significant difference (*p* < 0.001).

Distinct sex‐based differences in body coloration were evident in *D. vela* (Figure 4). Males exhibited significantly higher luminance in dorsal light markings (*t* = −5.619, *p* < 0.001), indicating brighter pigmentation in these regions. Conversely, females displayed significantly higher luminance in dorsal dark markings (*t* = −5.619, *p* < 0.001), as well as greater chroma values in both the throat (*t* = −2.623, *p* = 0.012) and lateral torso (*t* = −3.324, *p* = 0.002), suggesting more saturated coloration. No significant sex differences were observed in abdominal coloration.

### 3.3 Sexual Dimorphism in Bite Force

**FIGURE 5:**
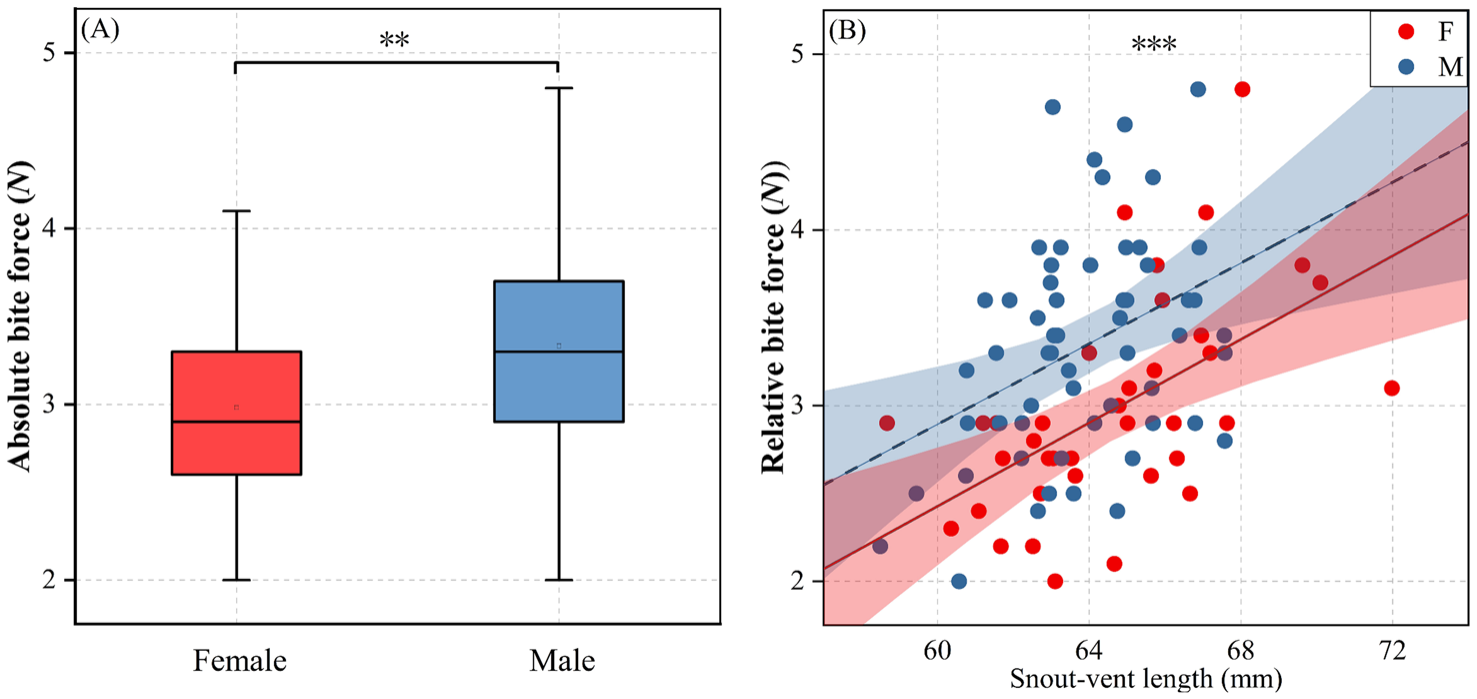
Comparison of bite force between the males and females. **Very significant difference (*p* < 0.01), ***Extremely significant difference (*p* < 0.001). (A) absolute bite force. (B) relative bite force with SVL controlled.

*Diploderma vela* exhibited pronounced male‐biased bite force. Males generated significantly higher absolute bite force than females (ANOVA, *F* = 7.214, *p* = 0.009, Figure 5A). This sex difference remained significant after controlling for SVL, with males maintaining greater bite force (MANCOVA, *F* = 15.233, *p* < 0.001, Figure 5B).

### 3.4 Sex‐Specific Correlations Between Bite Force and Phenotypic Traits

**FIGURE 6:**
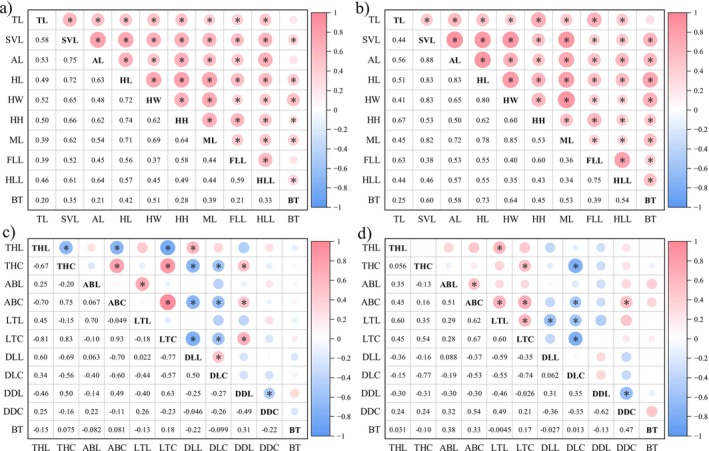
Correlation matrix of phenotypic traits (morphology and coloration) and maximum bite force. Panels (a, b) show the correlation tests between morphological traits and bite force in males and females, respectively. Panels (c, d) show the correlation tests between body coloration and bite force in males and females, respectively. ABC, abdomen chroma; ABL, abdomen luminance; DDC, dorsal dark markings chroma; DDL, dorsal dark markings luminance; DLC, dorsal light markings chroma; DLL, dorsal light markings luminance; LTC, lateral torso chroma; LTL, lateral torso luminance; THC, throat chroma; THL, throat luminance. For morphological abbreviations, refer to the 2. Materials and Methods section. Values represent correlation coefficients (*R*). Asterisks (*) indicate significant correlations (*p* < 0.05).

Correlation analysis revealed sex‐specific associations between morphological traits and bite force. In males, bite force was significantly positively correlated with SVL, HL, HW, HH, ML, and HLL (Figure 6a, *p* < 0.05). In females, all morphological traits except TL were significantly correlated with bite force (Figure 6b, *p* < 0.05).

**FIGURE 7:**
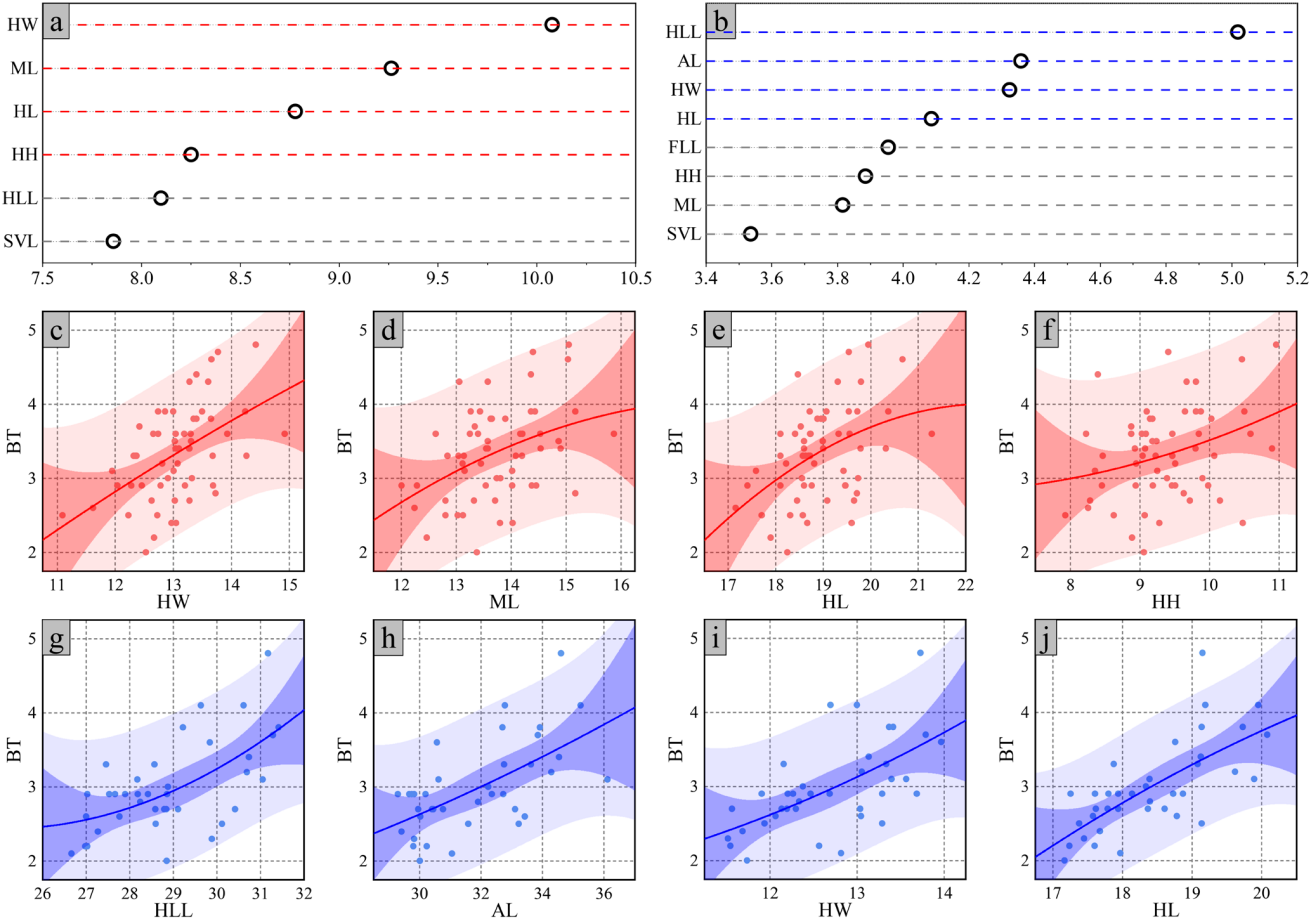
Variable importance of morphological traits for bite force based on random forest models and graphical illustrations of their relationships. Panels (a, b) show the variable importance values of morphological traits for males and females, respectively, with the X‐axis indicating the Mean Decrease Gini index. (c–f) and (g–j) illustrate the relationships between morphological traits and bite force for males and females, respectively. All abbreviations are provided in the 2. Materials and Methods section.

Random forest models further identified sexual dimorphic patterns of variable importance for bite force. For males, the key predictors in descending order of importance were HW, ML, HL, and HH (Figure 7a). Conversely, for females, HLL was the most influential, followed by AL, HW, and HL (Figure 7b). These sex‐specific predictive relationships are visualized in Figure 7c–j.

In contrast to morphology, body coloration showed no significant correlation with bite force in either sex (Figure 6c,d; *p* > 0.05), although the chroma of dorsal dark markings in females was significantly negatively correlated with SVL (*p* < 0.05, Table A1 of Appendix 1).

## 4 Discussion

Sexual dimorphism is a widespread phenomenon across diverse animal taxa (Zhong et al. 2017; Andre et al. 2018; Pincheira‐Donoso et al. 2020), frequently manifesting in multiple dimensions, including body size, morphological traits, coloration, and functional performance such as bite force (Herrel et al. 2018; Pincheira‐Donoso et al. 2020; Stuart‐Fox et al. 2020). In this study, we used systematic measurements and statistical analyses to investigate sexual dimorphism in the morphological and coloration traits of *D. vela* and to assess the relationships between these phenotypic traits and bite force. Our results revealed that *D. vela* exhibits significant sexual dimorphism across multiple morphological and coloration traits, which is consistent with our initial hypotheses. Furthermore, the correlations between morphological traits and bite force were sexually dimorphic, and the key predictors of bite force also differed between the sexes. Interestingly, however, coloration was not significantly correlated with bite force in either sex, contradicting our prediction and highlighting a potential mismatch between chromatic signals and functional performance.

Regarding morphological dimorphism, although SSD in lizards reflects a complex interplay of evolutionary forces, with documented patterns ranging from male‐biased and female‐biased to monomorphic across taxa (Schwarzkopf 2005; Cox et al. 2009; Herrel et al. 2010). Nevertheless, all existing studies on *Diploderma*, including the present one, consistently report a pronounced male‐biased pattern (Kuo 2009; Xiong et al. 2022; Li et al. 2025). Li et al. (2025) suggested that in *D. batangense*, males exhibit more frequent aggressive behaviors both within and outside the breeding season, which they attributed to resource scarcity in hot‐dry valley habitats and a reproductive strategy involving forced mating. We agree with this interpretation and propose that it also explains our findings, as extensive evidence indicates that larger body size, head dimensions, and limb structures confer significant advantages in resource competition and forced mating (Herrel et al. 2010; Hierlihy et al. 2013; Rosenthal 2017). Indeed, we frequently observed aggressive interactions among male *D. vela* in the field (unpublished data). Notably, multivariate analyses controlling for SVL revealed a contrasting female‐biased pattern in AL. Despite having similar SVL, females possessed significantly longer AL, consistent with the fecundity advantage hypothesis, which posits that increased abdominal volume enhances reproductive capacity by accommodating larger clutch size (Pincheira‐Donoso and Hunt 2017). Analysis of growth rates further revealed significant sexual divergence in TL in *D. vela*. We initially hypothesized that male tail length might be more critical than that of females in signaling individual performance, as documented in *Phrynocephalus vlangalii* (Peters et al. 2016; Wu et al. 2018). However, the subsequent finding that TL was not correlated with bite force in either sex did not support this idea. A fuller understanding of the ecological significance of these sex‐based differences in tail growth will need more detailed behavioral observations and analyses.

Coloration and ornamentation are critical phenotypic traits that mediate ecological communication, with their evolution shaped by competing selective pressures, including sexual selection, thermoregulation, predator avoidance, and physiological trade‐offs (Bu et al. 2020; Perez‐Martinez et al. 2020). While chromatic signals often convey complex, multimodal information—as seen in *Lacerta schreiberi*, where male dorsal coloration simultaneously reflects dominance and immune status (Martín and López 2009)—their functional outcomes exhibit taxon‐specific and context‐dependent complexity. Intraspecific divergence in coloration is further intensified by sex‐specific selection pressures and differential resource demands. This complexity is exemplified in *D. vela*, which displays an atypical pattern of sexual dichromatism. Unlike other mountain dragons where males bear vividly pigmented throats that function as dominance signals in competitive encounters (de Pérez I Lanuza et al. 2013; Wang et al. 2021), male *D. vela* lizards showed subdued throat coloration and reduced chromatic contrast relative to females. This reversed pattern of sexual dimorphism is rare among lizards and has only been reported in a few species, such as *Shinisaurus crocodilurus* (Qiu et al. 2022). However, in contrast to the functionally rich throat coloration of *Shinisaurus crocodilurus* , throat color in *D. vela* showed no significant association with body size or other performance‐related traits (Table A1 of Appendix 1). Instead, the most prominent sex‐linked color difference in *D. vela* occurred on the dorsum, where dorsal light markings in males were correlated with body size (Table A1 of Appendix 1). We speculate that these dorsal light markings may serve a signaling function analogous to the brightly colored throats of other mountain dragons, though this hypothesis requires further empirical validation with more extensive datasets. More importantly, whether this distinct phenotypic pattern is linked to adaptation to specific habitats and to divergent evolution among closely related species remains an open question, which will be the primary focus of our future research.

Bite force represents a key functional trait in lizards, directly mediating ecological performance across multiple behavioral contexts, including prey capture, territorial aggression, and mating competition (Lailvaux et al. 2004; Chazeau et al. 2013; Sagonas et al. 2014; Herrel et al. 2018; Naretto et al. 2022). In our study, *D. vela* exhibited significant sexual dimorphism not only in bite force itself, but also in the morphological traits correlated with it. To reduce multicollinearity with body size, we used random forest models for variable selection. Results confirmed that head morphology is the primary determinant of bite force, consistent with most previous studies (McBrayer and Anderson 2007; Herrel et al. 2007; Sagonas et al. 2014; Naretto et al. 2022). Previous work suggests that cranial morphology—particularly head size and shape—typically governs bite force through biomechanical optimization such as enhanced muscle leverage and expanded cross‐sectional area (Deeming 2022; Naretto et al. 2022). However, the strength of this relationship varies across taxa and between sexes (Li et al. 2025). Accordingly, our findings revealed that the specific head dimensions influencing bite force differ between the sexes: HW and ML were the primary predictors for males, whereas HW and HH were most influential for females. Increased HW likely provides greater volume for the attachment of enlarged temporal muscles, thereby enhancing bite force for effective prey immobilization (Herrel et al. 2005). The sex‐specific roles of ML and HH can be interpreted through structure–function relationships. In males, the jaw musculature and skeletal system are closely related to bite force, and it is clear from the principle of leverage that the larger the male force arm (ML), the greater the bite force generated (Herrel et al. 2007; Herrel et al. 2010). As noted by Li et al. (2025), this mechanical advantage is critical for males during resource competition and forced mating. Conversely, in females, bite force was associated with HL, suggesting adaptation for routine feeding efficiency (Herrel et al. 2001). These divergent trajectories underscore sex‐specific functional trade‐offs in cranial evolution, with males evolving structural weaponization and females maximizing resource‐processing capacity.

A particularly intriguing finding was that, in females, HLL was the strongest predictor of bite force. We hypothesize that this reflects functional integration between the locomotor and feeding systems, potentially improving foraging efficiency (Norberg and Norberg 2023). Such synergistic evolution between these systems could further increase predation success (Higham et al. 2025). However, we currently lack sufficient dietary data to test this hypothesis, despite multiple field observations of *D. vela* preying on orthopterans. Future studies should collect more comprehensive dietary, habitat, and behavioral data to clarify the ecological significance of these sexually dimorphic traits in *D. vela*.

In summary, *D. vela* exhibits pronounced sexual dimorphism in morphology, coloration, and the relationships between these phenotypic traits and bite force. These differences reflect the species' flexible phenotypic responses to varying reproductive and ecological pressures. However, a critical concern is the potential threat posed by hydropower development within its habitat. Habitat degradation not only alters the original vegetation, reducing the cryptic environments that match their dorsal markings but may also lead to shifts in dietary composition. Phenotypic traits associated with foraging may not respond rapidly to such anthropogenic changes. Although the modular nature of these traits might facilitate population persistence amid environmental variability, active conservation measures remain crucial for the long‐term survival of this species.

## Author Contributions

**Songwen Tan:** conceptualization (lead), data curation (lead), formal analysis (supporting), methodology (equal), validation (equal), visualization (supporting), writing – original draft (equal), writing – review and editing (equal). **Ling Li:** conceptualization (supporting), data curation (supporting), formal analysis (lead), methodology (equal), software (lead), validation (equal), writing – review and editing (equal). **Wei Gao:** conceptualization (supporting), data curation (supporting), formal analysis (lead), investigation (equal). **Guocheng Shu:** conceptualization (supporting), data curation (supporting), validation (equal), writing – review and editing (equal). **Peng Guo:** formal analysis (supporting), investigation (supporting), writing – review and editing (equal). **Yayong Wu:** conceptualization (lead), data curation (lead), formal analysis (supporting), funding acquisition (supporting), investigation (supporting), methodology (supporting), project administration (lead), software (supporting), visualization (supporting), writing – original draft (equal), writing – review and editing (supporting).

## Conflicts of Interest

The authors declare no conflicts of interest.

## Data Availability

All raw data and analysis code are stored in Dryad (DOI: 10.5061/dryad.44j0zpcvx).

## Appendix 1

**TABLE A1:** Correlation between body coloration and snout‐vent length in *D. vela*.

Region	Males (*n* = 17)		Females (*n* = 19)	
	Luminance	Chroma	Luminance	Chroma
Throat	0.314	−0.104	−0.137	0.347
Abdomen	0.286	0.116	−0.273	0.073
Lateral portions	0.276	0.122	−0.101	0.256
Dorsal light markings	0.29	0.218	−0.207	0.03
Dorsal dark markings	−0.17	−0.193	0.386	−0.493*

*Note:* Values are correlation coefficients (*R*), with **p* < 0.05 indicating significance.
